# Dentists’ Knowledge, Attitudes, and Awareness of Infection Control Measures during COVID-19 Outbreak: A Cross-Sectional Study in Saudi Arabia

**DOI:** 10.3390/ijerph17239016

**Published:** 2020-12-03

**Authors:** Ruba M. Mustafa, Ruwaida Z. Alshali, Dalea M. Bukhary

**Affiliations:** 1 Department of Conservative Dentistry, Faculty of Dentistry, Jordan University of Science and Technology, Irbid 22110, Jordan; 2 Oral and Maxillofacial Prosthodontics Department, Faculty of dentistry, King Abdulaziz University, Jeddah 21589, Saudi Arabia; ralshali@kau.edu.sa (R.Z.A.); dbukhary@kau.edu.sa (D.M.B.)

**Keywords:** coronavirus, dentists, surveys and questionnaires, Saudi Arabia, pandemics

## Abstract

As antiviral vaccines are still pending for the COVID-19 disease, improving dentists’ knowledge and prevention measures is important. This study aimed to assess dentists’ knowledge, attitude, and perception of COVID-19 in Saudi Arabia during the early outbreak period. In addition, infection control measures for dental setting were also assessed. Online questionnaire was distributed to dentists in different regions of Saudi Arabia when COVID-19 outbreak in Saudi Arabia was at its beginning. The questionnaire was assessing demographic variables, knowledge, attitude, risk perception, and preparedness towards COVID-19. Questions regarding infection control measures were also included. The correct incubation period of the virus was recognized by 43% of participants. Fever, cough, and shortness of breath were the mostly recognized symptoms for COVID-19 (98.9%, 95.5%, and 93.3% respectively). Participants in age groups ≥60, 50–59, and 20–29 years old were more likely to perceive COVID-19 as a very dangerous disease compared to 30–39 and 40–49 age groups. Dentists in Saudi Arabia showed satisfactory knowledge and positive attitude towards COVID-19. Improving dentists’ level of knowledge could be achieved through increasing their accessibility to materials provided by dental health care authorities, which specifies the best and safest approaches for dealing with patients during and after the outbreak.

## 1. Introduction

Novel coronavirus (2019-nCoV), also called (SARSCoV-2), caused an outbreak of pneumonia in late 2019 in Wuhan, China, which was confirmed by the Chinese Center for Disease Control and Prevention [1,2]. It was considered more infectious than severe acute respiratory syndrome coronavirus (SARS-CoV) and Middle East respiratory syndrome coronavirus (MERS-CoV) due to the rapidly increasing number of cases and evidence of human-to-human transmission with worldwide deaths [1]. The coronavirus family has been shown to cause multiple respiratory diseases, ranging from mild common cold to life threatening pneumonia, organ failure, and death [2].

This nosocomial transmission occurs most commonly through infectious saliva-associated respiratory tract secretions through two routes—direct or indirect. Direct transmission could be through a cough, sneeze, or droplet inhalation, while indirect transmission could be through contact with oral, nasal, or eye mucous membranes [3]. However, other possible routes of transmission were reported, such as fecal–oral transmission. This renders dental health providers to be at extremely high risk of infection from the SARS-CoV2 virus due to the nature of their work, being in direct and close contact with their patients [4]. Additionally, routine dental treatment procedures usually involve the use of instruments such as ultrasonic scalers, air-water syringes, and air turbine headpieces that become contaminated with the patients’ saliva and blood, and subsequently generate infectious aerosols and droplets in the workplace [3].

The typical clinical symptoms of COVID-19 reported by patients include fever, dry cough, breathing difficulty, and headache [2]. On the other hand, the majority of the cases remain asymptomatic or may demonstrate only mild symptoms similar to flu, seasonal allergy, and upper respiratory tract infections [5]. The symptoms start typically to appear after an estimated incubation period of 2–4 days [6], however this period may reach up to 24 days [7].

There is no approved antiviral treatment against COVID-19 at present; therefore, the only action for health care professions is to provide supportive care and follow preventive measures to prevent the spread of the infection [8]. During this pandemic period, guidelines are required that may serve to decrease the nosocomial transmission of COVID-19 [3,4] and prevent the possibility of recurrence of another outbreak [9]. The World Health Organization and Center for Disease Control and Prevention provided prevention recommendations and precautions for COVID-19 crisis [8]. On the 16th of March 2020, the American Dental Association recommended only emergency and urgent dental treatments and postponed any other routine and non-urgent dental treatments [10].

The global spread of COVID-19 led the World Health Organization (WHO) to reclassify it from an epidemic to a pandemic outbreak [11]. In April 2020, the WHO reported 533,416 laboratory-confirmed cases, 123,268 recoveries, and 24,110 deaths [12]. In November 2020, nearly 46 million cases and 1.2 million deaths had been reported worldwide. Europe showed half of the global new cases (over 1.7 million cases), while the highest numbers of deaths were reported from both Europe and the Americas, reaching over 17,000 new deaths [13]. The first case reported in Saudi Arabia was on 2 March 2020 [12]. In July 2020, Saudi Arabia reported the second largest number of cases, similar to the Eastern Mediterranean level [14,15]. By November 2020, the cumulative number of cases recorded in Saudi Arabia was 347,282, with 5402 deaths [13].

In Saudi Arabia, Covid-19 is the second outbreak that has affected the Middle East region, following MERS-CoV, which was reported in 2012 [16]. Since the first COVID-19 case was announced on the 2nd of March 2020 in Saudi Arabia, the country has implemented policies such as the suspension of school and work places. The Ministry of Health announced general health guidelines to combat the COVID-19 pandemic early in February 2020 [17]. In March 2020, it was announced that health care would be limited to emergency cases only, including dental care [17]. No specific dental health care guidelines to combat COVID-19 had been released by the Ministry of Health at the time of the study. In June 2020, the Ministry of Health released guidelines only for emergency dental procedures [18]. Recently, in October 2020, reopening guidelines were released for dental services [19]. During this global health crisis, the role of dental personnel including dentists and assistants is crucial in bringing awareness of COVID-19 to each other and to the community. Thus, it is very important that dental personnel have a high level of knowledge about COVID-19 and a positive attitude towards infection control measures and the seriousness of the disease. The aim of this study is to assess the awareness and knowledge of dentists in Saudi Arabia towards COVID-19 and infection control measures in the dental setting. This should identify any gaps in their knowledge, which would help to inform future planned interventions to improve dentists’ knowledge and awareness for better health education. In addition, data obtained from this study would act as a baseline for future studies aiming to assess and compare the aforementioned attributes at different times during the pandemic with the early period. The null hypothesis of the current study is that there would be no significant correlation between the demographic variables of dentists in Saudi and the different variables of knowledge, attitude, awareness of infection control, and perception of COVID-19 during the early pandemic outbreak period.

## 2. Materials and Methods

This study was a cross-sectional study using a non-probability convenience sampling method. This method allowed easy access to participants and quick data collection, as the period for data collection was restricted to the early period of the COVID-19 outbreak. Ethical approvals were obtained from the Institutional Review Board at Jordan University of Science and Technology (Ref. No.: 91/132/2020) and at King Abdulaziz University (Ref. No.:038-04-20). The target population comprised registered dentists in the Kingdom of Saudi Arabia. The sample size was obtained using a random sampling technique formula (Slovin’s formula). Calculations were based on the total number of licensed dentists in Saudi Arabia (16,887 dentist), as was reported in a study by Al Baker et al. (2017) [20]. The study power was set to 90% with a margin of error of 5%, which resulted in a minimum sample size of 265 dentists.

A Google Forms anonymous survey was distributed to around 600 dentists through WhatsApp groups of general and specialized dentists and through individual dentists’ Facebook accounts on the 17th of March and discontinued on the 3rd of April 2020. Participation was voluntary and the information provided by the participants was treated confidentially. Participants were free to stop answering the survey questions at any point.

### 2.1. Questionnaire Components and Assessment

The survey questions were adapted with permission from a previous study by Kader et al. (2020) [21] and constituted 26 questions in three sections. The first section assessed demographic variables (6 questions), including age, gender, education, work sector, place of practice, years of practice, and training courses or lectures attended for infection control in general or for COVID-19. In the second section, knowledge about COVID-19 (7 questions in total) was investigated, including the incubation period, symptoms (2 items), transmission, and protection measures (3 items). Finally, risk perception and preparedness for the disease (11 questions) were investigated (Appendix A). The questionnaire was assessed for validity by three experienced reviewers and the reliability was assessed through a pilot study using participants (*n* = 30) who were not included in the sample. The face validity of the questionnaire was assessed based on the relevance and structure of the questions by three experienced researchers who are familiar with the topic. A pilot study was run during early March 2020, which involved collecting responses from 30 Saudi dentists who were not included in the study. The responses were collected twice from participants with a one-week interval. A test–retest reliability assessment was performed using Cronbach’s alpha, giving a value of 0.89, which indicated high reliability.

### 2.2. Statistical Analysis

The data were analyzed using IBM Statistical Package for the Social Sciences (SPSS) version 25 (IBM Corporation, New York, NY, USA). Descriptive statistics were obtained and a chi-square test was employed to assess the relation between different categorical variables. The significance level was set at *p* ≤ 0.05.

## 3. Results

### 3.1. Demographic Data for Participants

A total of 269 responses were collected in the current study. Demographic data for the participants are summarized in Table 1, showing that 75.5% of the participants (203) had completed a master or residency program in dentistry. Regarding infection control in dentistry, 68% (183) had received training, while only 24.9% (67) had received training or attended educational courses about COVID-19.

### 3.2. Participants’ Knowledge of COVID-19

Regarding correct knowledge about the incubation period of the virus, 43.9% (118) answered this correctly (2–14 days), 44.6% (120) selected 7–14 days, 7.4% (20) selected 7–21 days, and 4.1% (11) selected 2–7 days.

Knowledge of different symptoms of the disease, situation and symptoms that should be considered to identify patients at risk of acquiring the virus, and infection control measures against COVID-19 are summarized in Table 2.

### 3.3. Participants’ Attitude and Risk Perception toward COVID-19

Regarding attitude and risk perception, 85.9% (231) agreed that personal protective equipment such as dental goggles, masks, and gloves are useful in protecting them from suspected COVID-19 patients. Furthermore, 93.3% (251) considered changing both masks and gloves as an important practice to decrease the possibility of transmitting infections from and to the patients. Here, 57.2% (154) perceived COVID-19 as very dangerous, 40.5% (109) as moderately dangerous, and the remaining perceived it as not dangerous. Additionally, 89.2% (240) believed that the COVID-19 pandemic does represent a serious public health issue.

A total of 57.2% (154) believed that COVID-19 symptoms require special treatment, while the remaining believed that they could resolve with time. All participants agreed that educating people about the disease is important to prevent its spread, and 86.6% (233) preferred to avoid working with patients suspected of COVID-19. Participants’ responses related to a number of attitude and preparedness for COVID-19 questions are summarized in Table 3.

**Figure 1:**
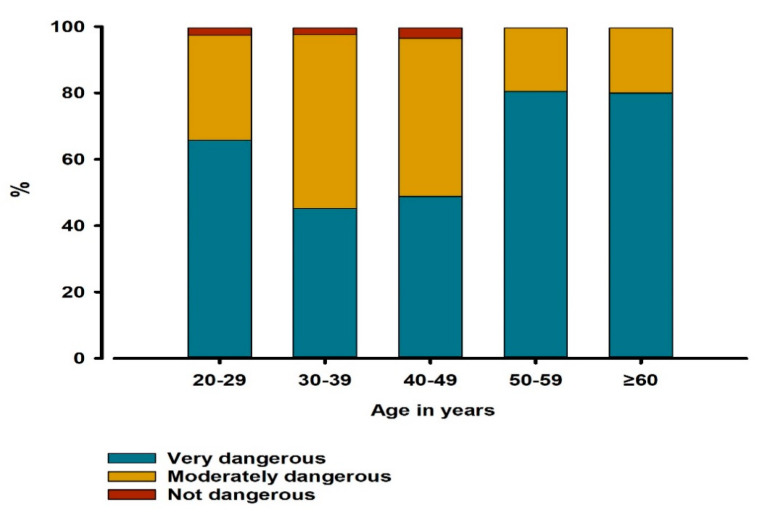
Distributions of different risk perception categories among different age groups of participants (*n* = 269). The relationship between age in years and risk perception of COVID-19 was assessed by chi-square test (*p* = 0.004).

The relationship between age and perception of COVID-19 was significant (*p* = 0.004). Participants in ≥60, 50–59, and 20–29 age groups were more likely to perceive it as a very dangerous disease (80.0%, 80.4%, and 65.8% of participants, respectively) compared to 30–39 and 40–49 age groups (45.2% and 48.8% of participants, respectively) (Figure 1). The relationship between knowledge about the incubation period and the work sector was insignificant (*p* = 0.067).

**Figure 2:**
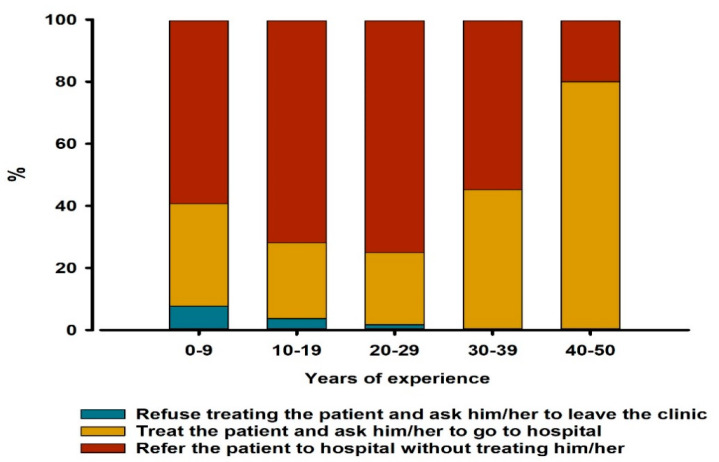
Distribution of different approaches for dealing with suspected COVID-19 patients among dentists with different years of practice (*n* = 269). The relation between years of practice and the attitude towards treating suspected COVID-19 patients was assessed by chi-square test (*p* = 0.024).

The relationship between the years of practice and attitude towards treating patients with symptoms of coughing or sneezing was significant (*p* = 0.024); dentists with 40–50 years of practice were more likely to treat those patients and ask them to go to hospital (80% of them). On the other hand, dentists with 20–29, 10–19, and 0–9 years of practice were more likely to refer a patient to the hospital without treating them (75%, 72%, and 59.3% of participants, respectively) (Figure 2). No significant relations between other categorical variables were demonstrated.

## 4. Discussion

The current study, which was conducted at the early stage of the COVID-19 outbreak, evaluated the general knowledge level, protection measures, and perceptions related to the COVID-19 outbreak among dentists in Saudi Arabia. The majority of the participants in this study were male dentists (60.6%), which is in accordance with recent statistics that showed that the percentage of licensed male dentists in Saudi Arabia is 61.06% [20].

In general, the knowledge level was satisfactory for dentists, in agreement with previous studies among health care workers (HCW) [22] and dental health providers for MERS-CoV [23]. However, the results showed that some gaps in the knowledge about the SARSCoV-2 virus do exist among the participants, as only 43.9% were able to determine the correct incubation period for the virus. This is in accordance with the findings of a recent study assessing the knowledge of Jordanian dentists about the SARSCoV-2 virus, where only 36.1% identified the correct incubation period [21]. In addition, a study among HCW in UAE showed that the correct incubation period was recognized by only 52% of participants [24]. The estimated incubation period was reported as up to 14 days [25], and in some rare cases it can be longer [7]. This asymptomatic period can be extremely dangerous for HCW, including dental staff, as the disease can spread before any symptoms are detected [4]. Therefore, it is recommended to increase the level of awareness in preventative measures in order to control its spread.

The current study showed that only about 25% of respondents had attended clinical training or lectures on COVID-19. This low percentage may be attributed to the period during which the study was conducted, when the outbreak in Saudi Arabia had just begun and the country was on lockdown. This finding suggests that educational measures should be considered in order to improve the knowledge level of dental staff. It is also highly recommended to increase dentists’ access to materials provided by health care authorities.

The satisfactory level of knowledge among the majority of the participants suggests that they were exposed to other information sources, including social media and the Internet. Nowadays, these sources are greatly effective and reachable at all times, increasing public awareness [26]. However, information delivered through these sources may not necessarily be accurate [27], resulting in misinformation about COVID-19 being spread among the public. Information delivered through social media had also been shown to result in the serious issue of international xenophobia [28]. In this perspective, information from reliable sources such as scientific health authorities and international health organizations are recommended to be the main informative sources [24].

The current study showed that participants have good knowledge about the mode of transmission and the clinical symptoms. This is important in identifying infected patients and controlling the spread of the disease [27]. This finding is consistent with findings from previous studies about MERS [27] and COVID-19 [21,24]. The most reported symptoms in this study were fever, cough, and shortness of breath.

All of the prevention measures—ranging from social distancing and hand washing to protective equipment, including surgical masks, face shields, gowns, and gloves—are important as protection measures for dental professionals in triage areas [3,29]. However, in dealing with suspected or diagnosed cases with SARS-CoV-2 infection, an N95 or equivalent or higher-level respirator is recommended in addition to the abovementioned protection measures [29]. However, infection control measures cannot be efficiently applied when the level of knowledge, perception, and compliance is poor among health care staff [30] and the population in general. In the current study, the perception of the prevention measures was positive, as 86% of participants perceived using protective equipment by dentists as necessary. This was positively reflected in their attitude towards practicing infection control measures, as 93% stated that changing both gloves and masks between patients is highly essential.

The increasing number of patients diagnosed positively for COVID-19, together with local and global reported deaths, turned COVID-19 into a hot topic on social media and among the public in Saudi Arabia and worldwide. Fear about infection transmission is increasing. This fact is reflected in our results, since the majority of participants (89%) considered COVID-19 as a serious public health issue. Moreover, around 57% perceived COVID-19 as a very dangerous disease and a similar percentage of participants agreed that it requires special treatment. To the best of our knowledge, we are still explicitly lacking antiviral treatment or a vaccine for COVID-19. As a result, infection control measures, early diagnosis, and meticulous supportive care are highly important [2]. On the other hand, it was reported that the majority of Jordanian dentists considered COVID-19 as a moderately dangerous issue, while one-third believed that COVID-19 was not a serious public health issue [21]. The difference in disease perception among Jordanian dentists and dentists in Saudi Arabia may be attributed to the times at which the studies were conducted—the study in Jordan was conducted when no local infection cases had been reported.

Interestingly, the current study showed that the percentage of dentists who perceived COVID-19 as a very dangerous issue was significantly higher among older dentists (above 50 years old age groups) than younger dentists. This can be explained by studies reporting that progression to the severe form of COVID-19 disease is higher in elderly patients [31]. In addition, the severe form of COVID-19 may cause severe acute respiratory illness, shock, and even death in some cases, especially patients with underlying comorbidities, such as hypertension, cardiovascular disorder, diabetes mellitus, chronic renal disease, chronic respiratory disorder, and immunosuppression [2]. As a result, older dentists perceived COVID-19 as a very dangerous issue, as older individuals are generally more prone to serious complications.

The current study showed a significant relationship between the years of practice and the dentists’ attitude towards treating patients with symptoms of coughing or sneezing. Dentists with more years of practice were shown to be more likely to treat those patients compared to dentists with less years of practice. Accordingly, the null hypothesis was partially rejected. This can be explained by the fact that the increased years of practice is associated with greater experience in dealing with infectious cases, since COVID-19 is the second outbreak in Saudi Arabia, with MERS-Cov having occurred in 2012 [16]. A previous study also suggested that precaution measures were positively associated with dentists having experience of longer than 10 years [23].

The majority of participants (86.6%) in the current study showed a negative response regarding working with suspected COVID-19 patients, which is in agreement with a previous study [21]. Moreover, 65.4% of participants would refer suspected patients to hospital without treatment. In addition, 85.1% would not allow their dental staff who demonstrate flu-like symptoms to work with patients. These findings may be attributed to different factors. First, the inherent attributes of clinical dental settings, such as the aerosol and droplets that are generated during dental procedures (e.g., from ultrasonic and sonic instruments, air–water syringes, and high-speed headpieces), result in viral particles traveling over a great distance and being suspended in the air for many hours, infecting dentists and dental assistants and contaminating surfaces in dental clinics [32]. Additionally, the close proximity between the dental staff and their patients puts dentists at extremely high risk of acquiring infectious diseases [32]. Furthermore, it has been shown that the standard infection control measures that are routinely applied during dental treatment were not adequate in preventing COVID-19 infection transmission [4]. Advanced precautionary procedures for dealing with suspected COVID-19 patients have been proposed [33], which are especially important with patients who are asymptomatic or at latent stage of the disease, when the virus sheds at a higher rate [7]. Another factor that may contribute to the negative responses of dentists towards dealing with suspected COVID-19 patients is the time during which the study was conducted (early during the outbreak). During this period, the dental management and infection control guidelines for COVID-19 from several dental associations all over the world were still not clear. Lastly, dentistry was classified as a very high-risk occupation for COVID-19 infection according to the Occupational Safety and Health Act Guidance, which was recently published [33]. In relation to the abovementioned factors, the American Dental Association recommended that routine and non-urgent dental treatments should be postponed to reduce disease transmission [10]. As antiviral vaccines are still pending, it would be highly useful to implement preventive strategies in the dental field. The prevention of dental caries through measures such as oral hygiene instructions, pit and fissure sealants, fluoride application, and diet analysis would be quite helpful in order to avoid or reduce the need for aerosol-generating restorative dental procedures in children. This could reduce the risk of viral infection transmission in the long term by reducing the need for advanced aerosol-generating dental procedures during the SARS-CoV-2 pandemic outbreak [34,35]. Moreover, the dental treatment procedures should be carried out under high standards of oral care and infection control, including patient examination prior to (telephone or telemedicine triage) or after attending a clinic (COVID19 questions form), clinical setting disinfection, medical wastes measures, reduction of the number of daily treated patients, and the use of personal protection during treatment [3,4,35]. Furthermore, using high-volume suction, rubber dam, and four-handed dentistry techniques could help in reducing the viral disease spread during dental treatment [4,29,35,36].

The majority of participants were aware of whom to contact in case of unprotected exposure to a known or suspected COVID-19 patient (82.9%) and what to do in case of encountering symptoms of COVID-19 (90.3%). This indicates the awareness of dentists in Saudi Arabia and their role in increasing population awareness regarding infection control and prevention measures. This finding is consistent with previous research findings among dentists in Jordan [21]. A number of cross-sectional studies have been conducted to assess awareness, knowledge, and infection control precautions among dental health providers in Saudi Arabia during the MERS-CoV outbreak, which have shown a high level of general awareness amongst dentists in the Saudi population [22,23,27].

Despite the promising results of this study, some limitations are worth mentioning. As this is a cross-sectional study, only associations can be presented, without cause–effect relationships. In addition, the self-reporting nature and the recall ability of the participants during completion of this survey should be considered. Moreover, this survey was conducted online among dentists during the early stage of the outbreak in Saudi Arabia, with a short period of time given for data collection while the majority were busy with the current outbreak news, which may have resulted in a limited sample size, in turn limiting the generalizability of the findings. Lastly, since the study was conducted during the lockdown, the online survey through social networks such as WhatsApp and Facebook was the only applicable option, which might have increased the occurrence of sampling errors. Fortunately, the penetration percentage for WhatsApp use and Facebook were reported ar around 73% and 66%, respectively, in Saudi Arabia in 2017 [37], which might have minimized the sampling bias risk. Further future studies, however, are recommended after a period of quarantine with a larger sample size that allows better generalization of the results.

## 5. Conclusions

In general, dentists in Saudi Arabia involved in the current survey showed satisfactory knowledge and a positive attitude towards COVID-19 during the outbreak. However, there is still scope for recommendations to improve the knowledge level amongst dental staff. In addition, it is recommended to increase the dentists’ access to materials provided by dental health care authorities and to specify the best and safest approaches when dealing with COVID-19 patients during and after the outbreak.

## Figures and Tables

**Table 1:** Demographic data for participants.

Demographic Variables	n	%
**Gender**		
Male	163	60.6
Female	106	39.4
**Age in years**		
20–29	38	14.1
30–39	84	31.2
40–49	86	32.0
50–59	46	17.1
≥60	15	5.6
**Years of practice**		
0–9	91	33.8
10–19	82	30.5
20–29	60	22.3
30–39	31	11.5
40–50	5	1.9
**Region of practice in Saudi Arabia**		
Central region	121	45.0
Northern region	8	3.0
Western region	54	20.1
Eastern region	15	5.6
Southern region	70	26.0
**Working sector**		
Academic	111	41.3
Private	30	11.2
Public	78	29.0
Military	37	13.8
Other	13	4.8

**Table 2:** Participants’ responses to knowledge questionnaire items.

Question	n	%
**Which of the following are symptoms of COVID-19?**		
Fever	266	98.9
Cough	257	95.9
Runny nose	74	27.5
Sore throat	171	63.6
Shortness of breath	251	93.3
Joint/muscle pain	118	43.9
Red eyes	19	7.1
Skin rash	9	3.3
Diarrhea	70	26.0
Vomiting	37	13.8
May present with no symptoms	121	45.0
**Which of the following should be considered to identify patients at risk of having COVID-19?**		
The presence of symptoms of diarrhea	24	8.9
The presence of symptoms of respiratory infection	246	91.4
History of travel to areas experiencing transmission of COVID-19	257	95.5
History of contact with possible infected patients	256	95.2
**Which of the following measures should be taken to prevent transmission from known or suspected COVID-19 patients?**		
Eat boiled and cooked food	92	34.2
Put facemask on known or suspected patients	237	88.1
Place known or suspected patients in adequately ventilated single rooms	211	78.4
All health staff members wear protective clothing	225	83.6
Avoid moving and transporting patients out of their area unless necessary	228	84.4
Frequently clean hands by using alcohol-based hand rub or soap and water	262	97.4

**Table 3:** Participants’ responses to attitude and preparedness questions.

Question	n	%
**Do COVID-19 symptoms often resolve with time and do not require any special treatment?**		
Yes	115	42.8
No	154	57.2
**Is it important to educate people about COVID-19 do prevent the spread of the disease?**		
Yes	269	100
No	0	0.0
**Do you prefer to avoid working with a patient who is suspected of COVID-19?**		
Yes	233	86.6
No	36	13.4
**What would you do in case a patient is sneezing or coughing in your clinic?**		
Refuse treating the patient and ask him/her to leave the clinic	11	4.1%
Treat the patient and ask him/her to go to hospital	82	30.5%
Refer the patient to the hospital without treating him/her	176	65.4%
**What do you think about asking patients to set far from each other, wearing masks in the waiting area and washing hands before getting on the dental chair?**		
Necessary and help to reduce disease transmission	253	94.1%
Not necessary and could cause panic	16	5.9%
**Would you allow any of your dental staff to work with patients if they have flu-like symptoms?**		
Yes	40	14.9%
No	229	85.1%
**What do you think about the dentist’s role in educating others about COVID-19?**		
Very significant	223	82.9%
Moderately significant	37	13.8%
Mildly significant	7	2.6%
Not significant at all	2	0.7%
**Do you know who to contact in case of unprotected exposure to a known or suspected COVID-19 patient?**		
Yes	223	82.9%
No	46	17.1%
**Do you know what to do if you have signs or symptoms suspected of COVID-19 infection?**		
Yes	243	90.3%
No	26	9.7%

## Appendix A

**A supplementary table including the survey questions**

**Dentists’ Knowledge, Attitude, and Awareness of Infection Control Measures during COVID-19 Outbreak: A Cross-Sectional Study in Saudi Arabia**
The concern regarding coronavirus is increasing and new cases are emerging all over the world. As a group of researchers, we are doing a short (<5 min) survey to assess the level of knowledge that we have about coronavirus. We request your kind support to participate in this survey and also recommend your colleagues in Saudi Arabia to participate in it. This survey is anonymous; the information you provide is only for educational and research purposes and it will be treated confidentially. Your participation is highly appreciated.
**Consent**
I agree to participate in this study ○ Yes ○ No
I. **Demographic and general characteristics**
1. Gender ○ Male ○ Female
2. Age group ○ 20–29 ○ 30–39 ○ 40–49 ○ 50–59 ○ ≥60
3. Years of practice ○ 0–9 ○ 10–19 ○ 20–29 ○ 30–39 ○ 40–50
4. Region of practice in Saudi Arabia: ○ Northern ○ Southern ○ Western ○ Eastern ○ Central
5. You are currently working in: ○ Academia ○ Private sector ○ Public sector ○ Military ○ Other
6. Have you completed a master/PhD or residency program in dentistry? ○ Yes ○ No
7. Do you have any infection control in dentistry? ○ Yes ○ No
8. Have you attended any training or received lectures regarding COVID-19? ○ Yes ○ No
II. **Knowledge**
9. What is the incubation period of corona virus? ○ 2–7 days ○ 2–14 days ○ 7–14 days ○ 7–21 days
10. Which of the following are symptoms of COVID-19? select all applicable ○ Fever ○ Cough ○ Runny nose ○ Sore throat ○ Shortness of breath ○ Joint/muscle pain ○ Red yes ○ Skin rash ○ Diarrhea ○ Vomiting ○ May present with no symptoms
11. How can COVID-19 be transmitted? Select all applicable ○ Via coughing and sneezing ○ Hans shaking ○ Touching surfaces as doorknobs and tables
12. Which of the following should be considered to identify patients at risk of having COVID-19? Select all applicable ○ The presence of symptoms of diarrhea ○ The presence of symptoms of respiratory infection ○ History of travel to areas experiencing transmission of COVID-19 ○ History of contact with possible infected patients
13. Which of the following measures should be taken to prevent transmission from known or suspected COVID-19 patients? select all applicable ○ Eat boiled and cooked food ○ Put facemasks on known or suspected patients ○ Place known or suspected patients in adequately ventilated single rooms ○ All health staff members wear protective clothing ○ Avoid moving and transporting patients out of their area unless necessary ○ Frequently clean hands by using alcohol-based hand rub or soap and water
14. Personal protective equipment such as dental goggles, FFP2 (KN95) masks, and gloves are useful in protecting me from a patient suspected to have COVID-19? ○ Disagree ○ Agree ○ Neutral
15. How important is to change both masks and gloves to decrease the possibility of transmitting infections to patients and to myself? ○ Very important ○ Important ○ Little important ○ Not important
III. **Risk perception and preparedness**
16. How do you perceive COVID-19? ○ Very dangerous ○ Moderately dangerous ○ Not dangerous
17. Do you believe COVID-19 is not currently a serious health issue? ○ Yes ○ No
18. COVID-19 symptoms often resolve with time and do not require any special treatment? ○ Yes ○ No
19. Is it important to educate people about COVID-19 to prevent the spread of the disease? ○ Yes ○ No
20. Do you prefer to avoid working with a patient who is suspected of COVID-19? ○ Yes ○ No
21. What would you do in case a patient is sneezing or coughing in your clinic? ○ Refuse treating the patient and ask him/her to leave the clinic ○ Threat the patient and ask him/her to go to hospital ○ Refer the patient to hospital without treating him/her
22. Do you think about asking patients to set far from each other, wearing masks while in the waiting room, and washing hands before getting in the dental chair? ○ Necessary and help to decrease disease transmission ○ Not necessary and could cause panic
23. Would you allow any of your dental staff to work with patients if they have flu-like symptoms? ○ Yes ○ No
24. What do you think about the dentist’s role in teaching others about COVID-19? ○ Very significant ○ Moderately significant ○ Mildly significant ○ Not significant at all
25. Do you know whom to contact in a situation where there has been an unprotected exposure to a known or suspected COVID-19 patient? ○ Yes ○ No
26. Do you know what to do if you have signs or symptoms suspected of COVID-19 infection? ○ Yes ○ No
